# Supplementary figures and images for: The read-through transcription-mediated autoactivation circuit for virulence regulator expression drives robust type III secretion system 2 expression in Vibrio parahaemolyticus
Source: PLoS Pathog. 2024 Mar 27;20(3):e1012094. doi: 10.1371/journal.ppat.1012094 (PMC10971746; doi:10.1371/journal.ppat.1012094)

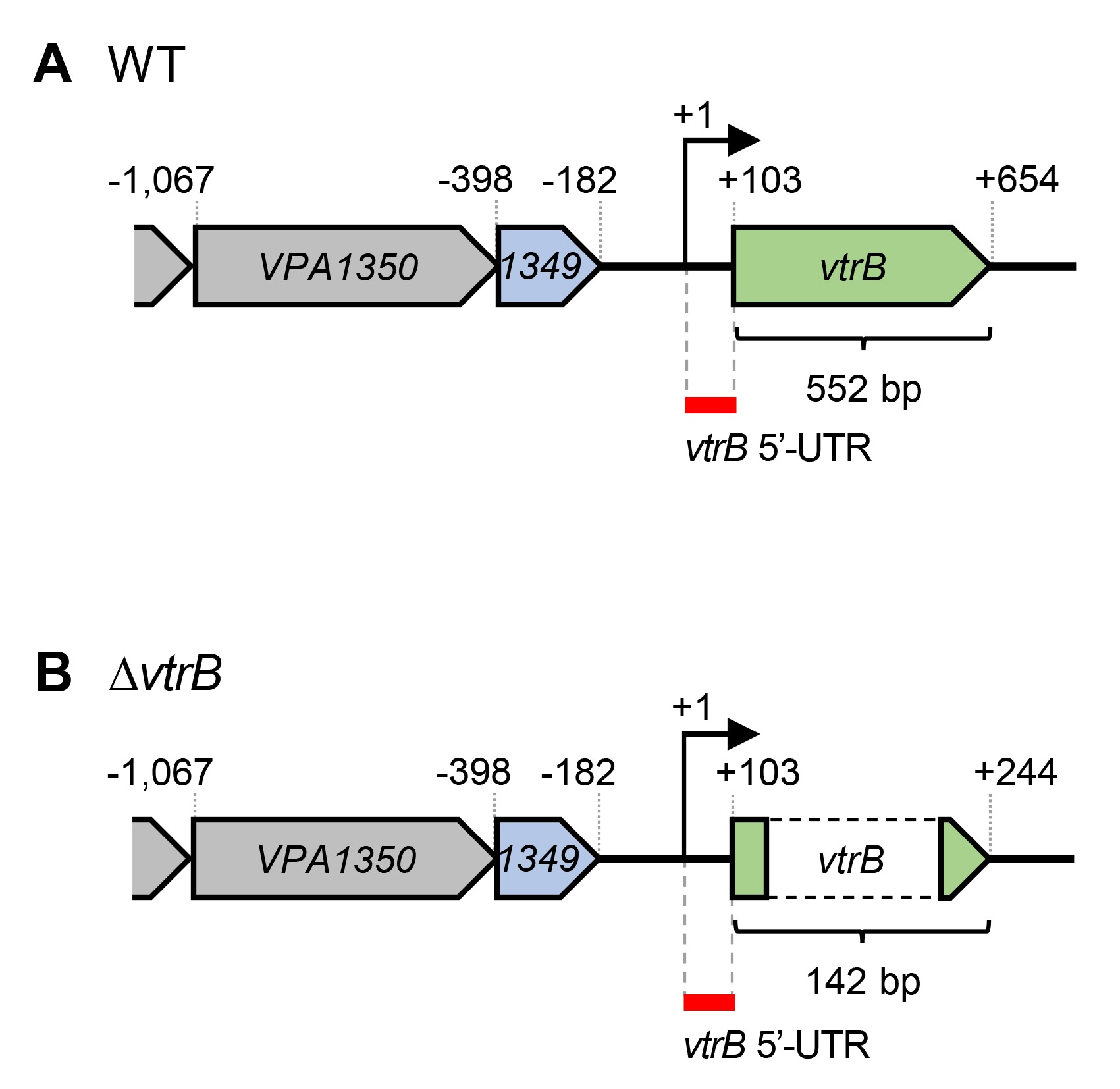

Supplement: S1 Fig — Schematic representation of the vtrB upstream region with adjacent genes in the WT (A) and ΔvtrB strains (B). The arrows indicate genes with their orientation. The nucleotide position is based on the transcriptional start site of vtrB (indicated as +1). The coding sequence of vtrB has a length of 552 bp, whereas the ΔvtrB strain contains a 410-bp deletion in the coding sequence. (TIF) [file ppat.1012094.s001.tif]

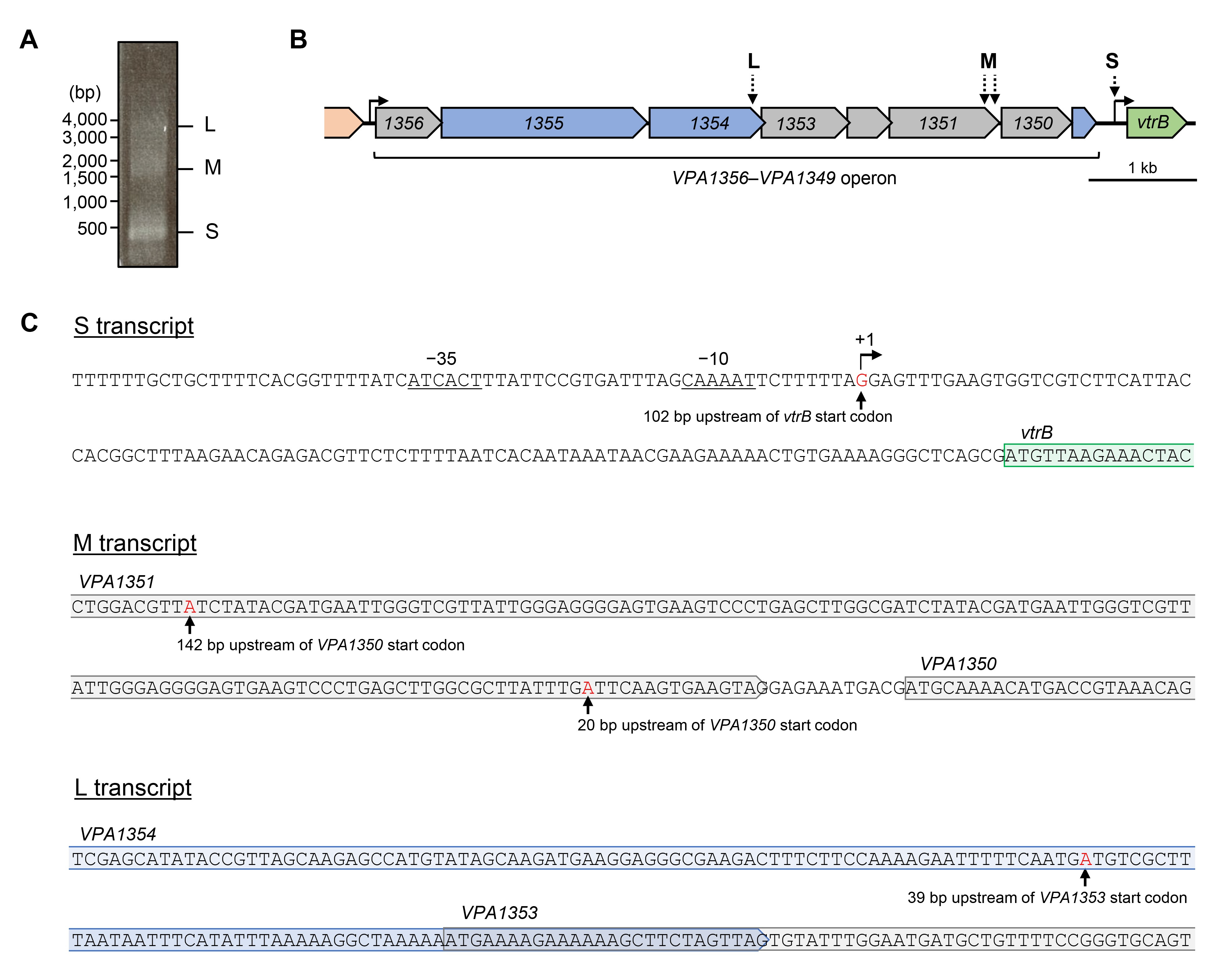

Supplement: S2 Fig — (A) Agarose gel electrophoresis of 5’-RACE PCR products. (B) Schematic representation of the position of the mapped 5’-end of each vtrB transcript from 5’-RACE PCR. (C) Nucleotide sequence around the 5’-ends of 5’-RACE products. The determined 5’-ends are indicated in red. The transcriptional start site of the vtrB is indicated as +1, and putative −35 and −10 elements are underlined. The shading indicates the region of coding sequences, and the names of the genes are shown above. (TIF) [file ppat.1012094.s002.tif]

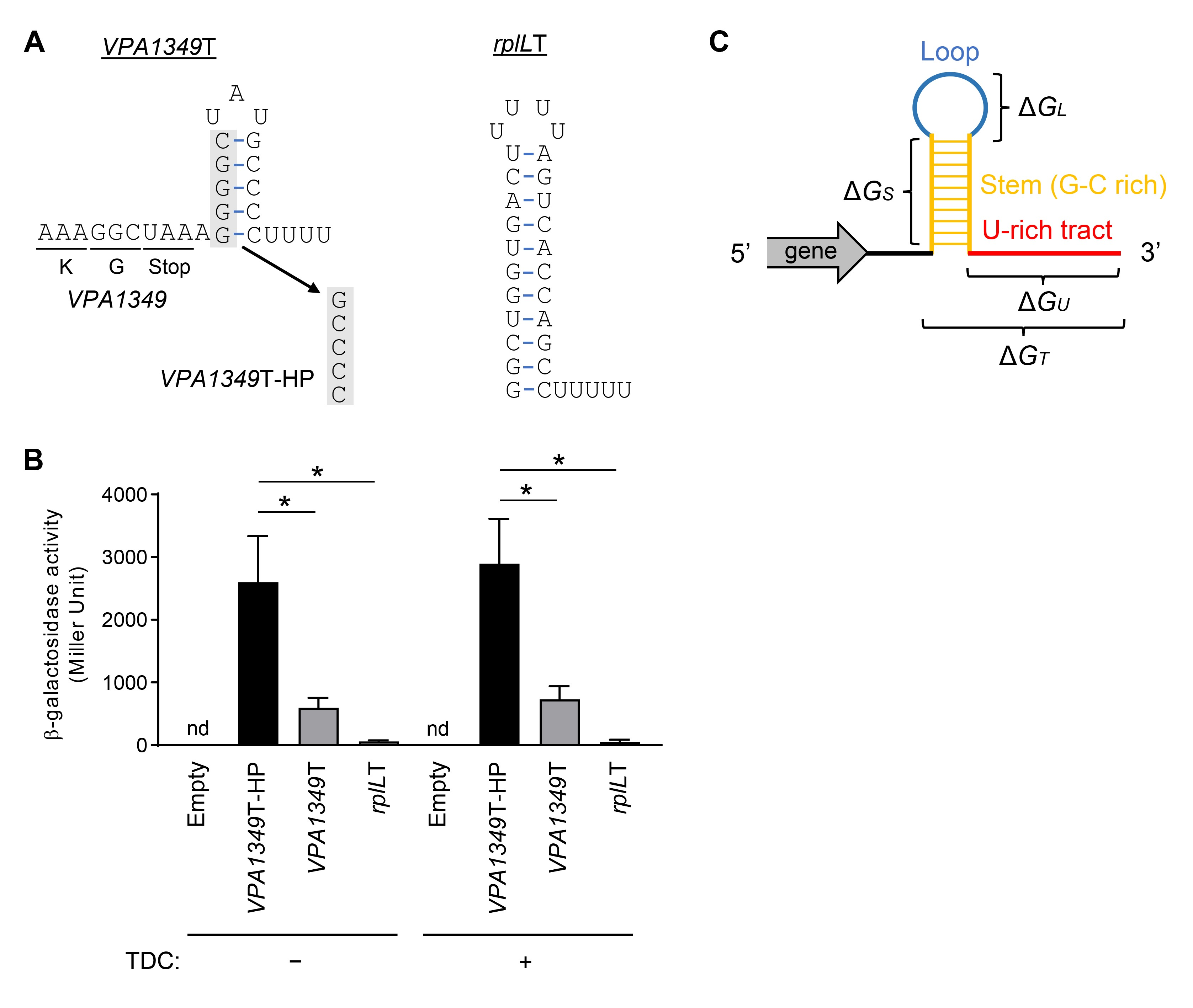

Supplement: S3 Fig — (A) The secondary structure of the VPA1349 terminator (VPA1349T) and the E. coli rplL terminator (rplLT) predicted using Mfold [22]. Gray shading indicates the substituted nucleotides (GGGGC > CCCCG) for disrupting the hairpin structure formation in VPA1349T. (B) Evaluation of the transcription termination ability using the terminator-fused lacZ reporters. The V. parahaemolyticus ΔvtrB strain harboring each reporter plasmid with VPA1349T, rplLT, or the VPA1349 terminator hairpin mutant (VPA1349T-HP) was grown in LB medium containing 0.3 M NaCl with or without TDC induction, and the β-galactosidase activity was monitored. The values show the means and error bars represent the SDs (n = 3). nd, not detected; *, p < 0.05, as revealed by one-way ANOVA followed by Dunnett’s multiple comparison test. (C) Schematic structure of the general intrinsic terminator (adapted from [50] with modifications). (TIF) [file ppat.1012094.s003.tif]

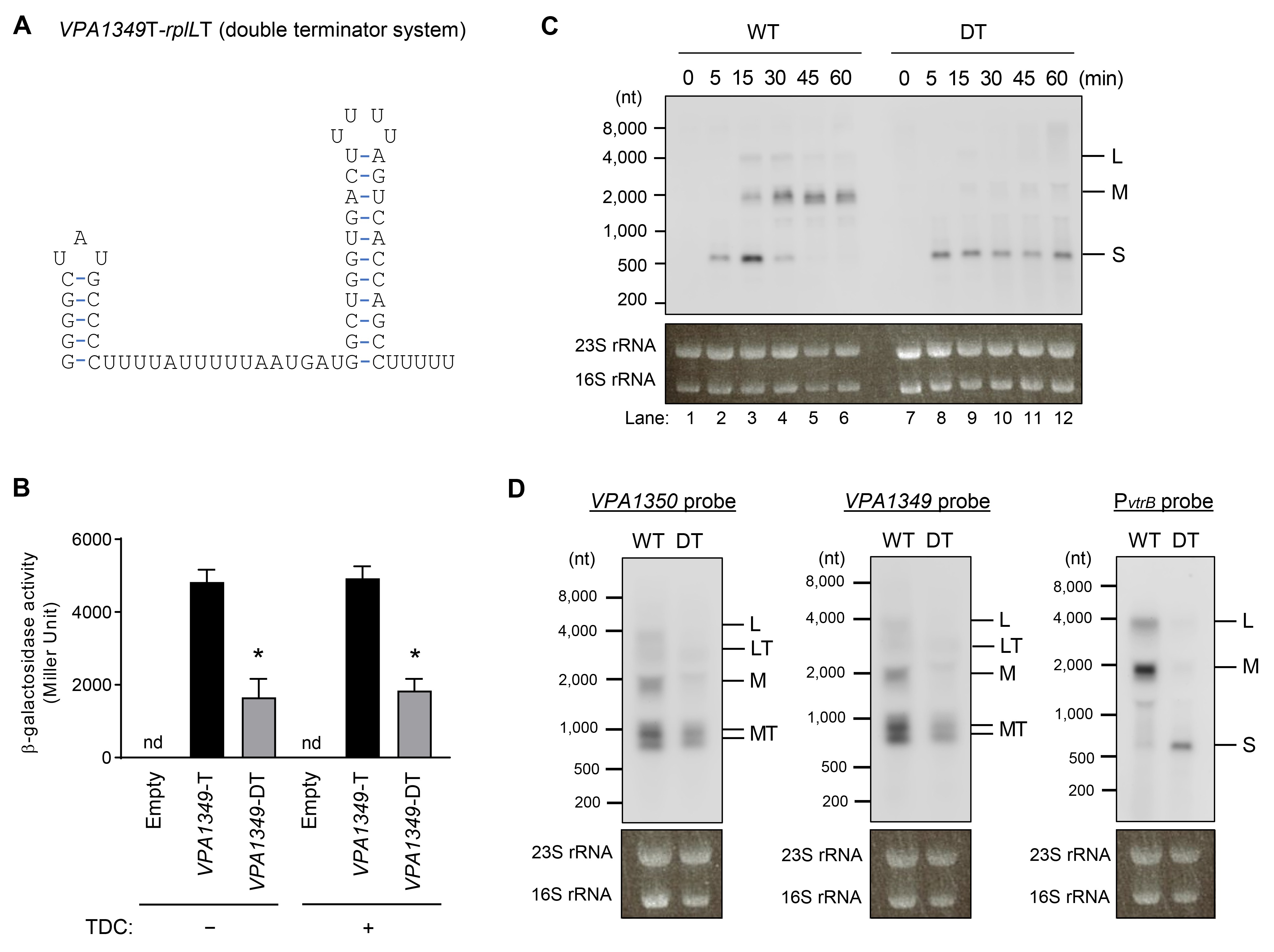

Supplement: S4 Fig — (A) The secondary structure of the double terminator system composed of VPA1349T and rplLT was predicted using Mfold [22]. (B) Transcription termination ability of the double terminator system. V. parahaemolyticus ΔvtrB with lacZ reporter plasmids containing the VPA1349 gene with VPA1349T or with the double terminator (VPA1349-DT) was grown in LB medium containing 0.3 M NaCl with or without TDC, and the β-galactosidase activity was evaluated. The values show the means and error bars represent the SDs (n = 3). nd, not detected; *, p < 0.05, compared with VPA1349-T by Student’s t test. (C) Effect of the double terminator on the vtrB transcript profile in V. parahaemolyticus. The WT and DT strains were grown to an OD600 of 0.8, and TDC was then added. RNA was extracted after 0, 5, 15, 30, 45, and 60 min of TDC induction, and northern blotting was performed using the PvtrB probe. L, L transcript; M, M transcript; S, S transcript. 23S rRNA and 16S rRNA served as loading controls. (D) Transcription termination at the double terminator. V. parahaemolyticus WT and DT strains were grown in LB medium containing 0.3 M NaCl to an OD600 of 0.8, and TDC was then added. After further incubation for 15 min, total RNA was extracted, and northern blotting was performed using the indicated probes: the VPA1350 coding region (left), the VPA1349 coding region (center) and PvtrB (right). L, L transcript; LT, L transcript terminated at VPA1349T or the double terminator; M, M transcript; MT, M transcript terminated at VPA1349T or the double terminator; S, S transcript. The data are representative of three independent experiments (C, D). (TIF) [file ppat.1012094.s004.tif]

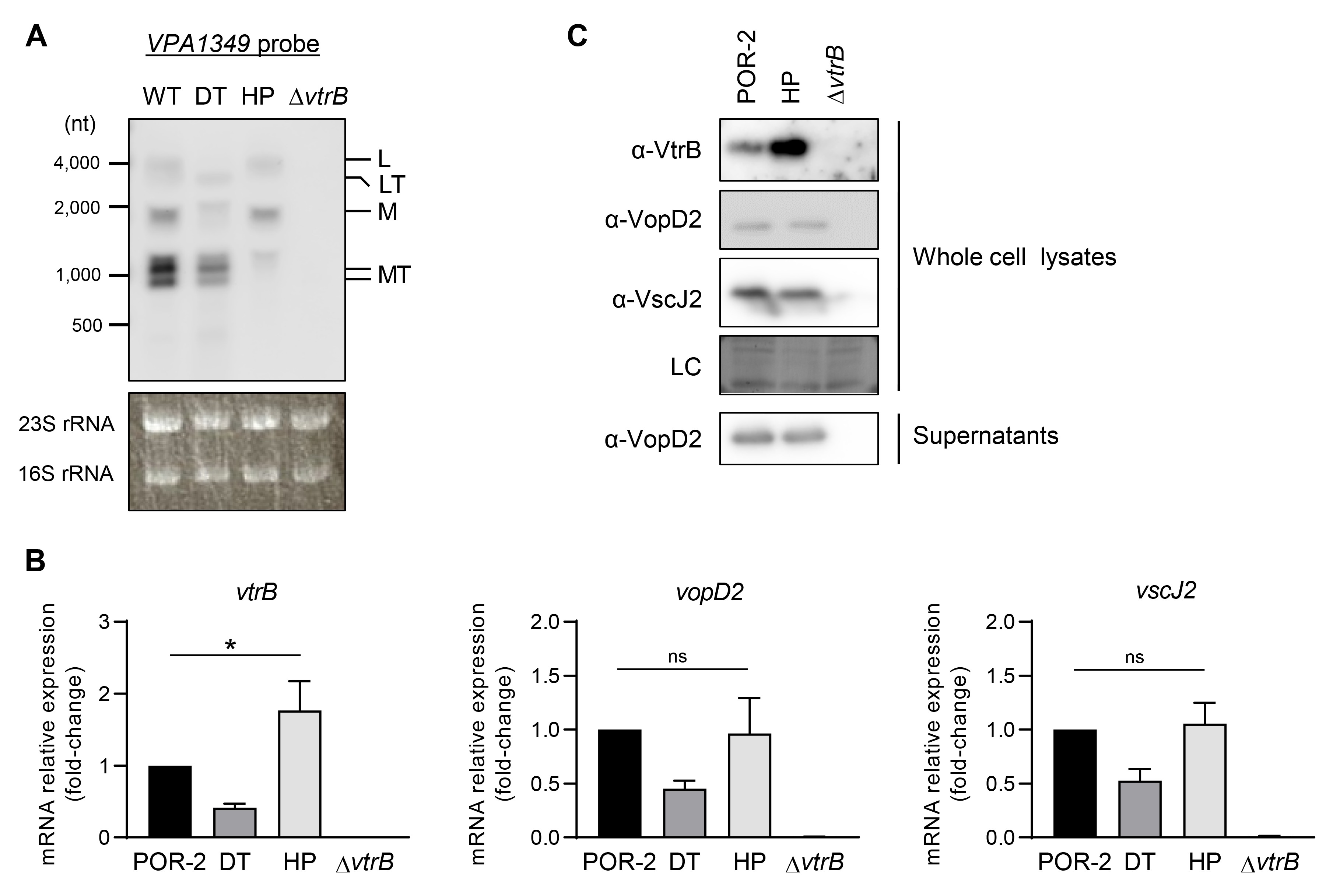

Supplement: S5 Fig — (A) V. parahaemolyticus strains WT, WT-derived DT (DT), ΔvtrB, and a strain carrying the hairpin mutation in VP1349T (S3A Fig: VP1349T-HP) on the chromosome of the WT strain (HP strain) were grown in LB medium containing 0.3 M NaCl to an OD600 of 0.8, and TDC was then added. After further incubation for 15 min, total RNA was extracted, and northern blotting was performed using the probe for the VPA1349 coding region. The HP strain was defective in transcription termination at the end of the upstream operon, as observed by the absence of L and M transcripts terminated at VP1349T. L, L transcript; LT, L transcript terminated at VPA1349T; M, M transcript; MT, M transcript terminated at VPA1349T. The data are representative of three independent experiments. (B) Effect of the hairpin mutation in VPA1349T on vtrB and VtrB-regulated gene expression. V. parahaemolyticus strains POR-2, POR-2 DT (DT), POR-2 HP (HP), and POR-2 ΔvtrB (ΔvtrB) were grown under the inductive condition, and total RNA was extracted from each culture once the culture reached an OD600 of 1. Relative expression of vtrB, vopD2, and vscJ2 with the housekeeping gene recA was analyzed by qRT–PCR. The values represent the means ± SDs from a minimum of three independent experiments. *, p < 0.05; ns, not significant, compared with POR-2 by Student’s t test. (C) Effect of the hairpin mutation in VPA1349T on the production of VtrB and T3SS2-related proteins. Bacterial whole-cell lysates and culture supernatants of V. parahaemolyticus POR-2, HP, and ΔvtrB strains grown under the inductive condition to an OD600 of 1.8 were analyzed by immunoblotting with the indicated antibodies. Whole-cell lysate proteins on the blotted membrane were visualized with Ponceau S staining for loading control (LC). (TIF) [file ppat.1012094.s005.tif]
