# Supplementary material for: The read-through transcription-mediated autoactivation circuit for virulence regulator expression drives robust type III secretion system 2 expression in Vibrio parahaemolyticus
Source: PLoS Pathog. 2024 Mar 27;20(3):e1012094. doi: 10.1371/journal.ppat.1012094 (PMC10971746; doi:10.1371/journal.ppat.1012094)
Supplement: S1 Table — (PDF) [file ppat.1012094.s006.pdf]

S1 Table. Gibbs free energy (ΔG) values of *VPA1349*T and *rplL*T, calculated using Mfold [23].

	ΔG_T (kcal/mol)	ΔG_S (kcal/mol)	ΔG_L (kcal/mol)	ΔG_U (kcal/mol)
*VPA1349*T	−8.80	−13.30	5.70	−1.2
*rplL*T	−21.60	−25.20	4.80	−1.2
