# Supplementary material for: The read-through transcription-mediated autoactivation circuit for virulence regulator expression drives robust type III secretion system 2 expression in Vibrio parahaemolyticus
Source: PLoS Pathog. 2024 Mar 27;20(3):e1012094. doi: 10.1371/journal.ppat.1012094 (PMC10971746; doi:10.1371/journal.ppat.1012094)
Supplement: S2 Table — (PDF) [file ppat.1012094.s007.pdf]

S2 Table. Bacterial strains used in this study.

Strains	Genotype/description	Reference
V. parahaemolyticus		
WT (RIMD2210633)	*tdh*- and T3SS2-positive clinical isolate; serotype O3:K6	[1]
Δ*vtrA*	*vtrA*-deleted strain derived from WT	[2]
Δ*vtrB*	*vtrB*-deleted strain derived from WT	[2]
Δ*vtrA* Δ*vtrB*	*vtrA*- and *vtrB*- deleted strain derived from WT	[2]
DT	*rplL*T-inserted strain derived from WT	This study
HP	WT-derived strain with *VPA1349*T-HP	This study
POR-2	TDH- and T3SS1-deficient strain derived from WT	[3]
POR-2 DT	*rplL*T-inserted strain derived from POR-2	This study
POR-2 HP	POR-2 derived strain with *VPA1349*T-HP	This study
POR-2 Δ*vtrB*	*vtrB*- deleted strain derived from POR-2	This study
E. coli		
DH5α	F– Φ80Δ*lacZ*M15 Δ(*lacZYA argF*)U169 *deoP recA1 endA1 hsdR17*(rK− mK−)	Laboratory collection
BW19851	F– *RP4-2*(*Km::Tn7,Tc::Mu-1*) Δ*uidA3::pir*+ *recA1 thiE1 hsdR17 creC510*	Laboratory collection

References

1. Makino K, Oshima K, Kurokawa K, Yokoyama K, Uda T, Tagomori K, et al. Genome sequence of *Vibrio parahaemolyticus*: a pathogenic mechanism distinct from that of *V. cholerae*. Lancet. 2003; 361: 743–749.
2. Kodama T, Gotoh K, Hiyoshi H, Morita M, Izutsu K, Akeda Y, et al. Two regulators of *Vibrio parahaemolyticus* play important roles in enterotoxicity by controlling the expression of genes in the Vp-PAI region. PLoS One. 2010; 5: e8678.
3. Park KS, Ono T, Rokuda M, Jang MH, Okada K, Iida T, et al. Functional characterization of two type III secretion systems of *Vibrio parahaemolyticus*. Infect Immun. 2004; 72: 6659–6665.
