# Supplementary material for: The read-through transcription-mediated autoactivation circuit for virulence regulator expression drives robust type III secretion system 2 expression in Vibrio parahaemolyticus
Source: PLoS Pathog. 2024 Mar 27;20(3):e1012094. doi: 10.1371/journal.ppat.1012094 (PMC10971746; doi:10.1371/journal.ppat.1012094)
Supplement: S3 Table — (PDF) [file ppat.1012094.s008.pdf]

S3 Table. Plasmids used in this study.

Plasmid	Description	Reference
pBAD18-Cm	P_{BAD} promoter, pBR322 *ori*, Cm^R	[1]
pBAD18-Cm-*vtrB*	pBAD18-Cm containing *vtrB* gene	[2]
pBAD18-Cm-*vtrA*	pBAD18-Cm containing *vtrA* gene	[3]
pCRII-TOPO	TA cloning vector, ColE1 *ori*, Km^R, Amp^R	Thermo Fisher Scientific
pYAK1	Suicide vector for gene replacement, *ori*R6K, Cm^R	[4]
pYAK1-DT	A derivative of pYAK1 for integration of *rplL*T downstream of *VPA1349*T	This study
pYAK1-HP	A derivative of pYAK1 for replacement of *VPA1349*T with *VPA1349*T-HP	This study
pHRP309	*lacZ* transcriptional fusion vector, Gm^R	[5]
pHRP309-$UP_{VPA1356}$	pHRP309 containing 112 bp upstream region of *VPA1356*	This study
pHRP309-$UP_{VPA1350}$	pHRP309 containing 304 bp upstream region of *VPA1350*	This study
pHRP309-P_{vtrB}	pHRP309 containing *vtrB* promoter (284 bp upstream of the *vtrB* start codon)	[3, 6]
pHRP309-*VPA1356*-$UP_{VPA1350}$	pHRP309 containing 5,724 bp downstream of the *VPA1356* start codon	This study
pHRP309-$UP_{VPA1356}$-$UP_{VPA1350}$	pHRP309 containing -112 bp to +5,724 bp of the *VPA1356* start codon	This study
pHRP309-$UP_{VPA1356}$-*VPA1349*	pHRP309 containing -112 bp to +6,609 bp of the *VPA1356* start codon	This study
pHRP309-$UP_{VPA1356}$-$DN_{VPA1349}$	pHRP309 containing -112 bp to +6,755 bp of the *VPA1356* start codon	This study
pHRP309-$UP_{VPA1356}$-P_{vtrB}	pHRP309 containing -112 bp to +6,893 bp of the *VPA1356* start codon	This study
pHRP309-$UP_{VPA1353}$	pHRP309 containing 318 bp upstream region of *VPA1353*	This study
pBAD-*VPA1349*T-HP-*lacZ*	pBAD18-Cm containing *VPA1349*T with hairpin mutation (GGGGC > CCCCG) and *lacZ* gene	This study
pBAD-*VPA1349*T-*lacZ*	pBAD18-Cm containing *VPA1349*T and *lacZ* gene	This study

S3 Table. **Plasmids used in this study** (continued)**.**

Plasmid	Description	Reference
pBAD-*rplL*T-*lacZ*	pBAD18-Cm containing *rplL*T and *lacZ* gene	This study
pBAD-*VPA1349*-T-*lacZ*	pBAD18-Cm containing *VPA1349* genes with *VPA1349*T, and *lacZ* gene	This study
pBAD-*VPA1349*-DT-*lacZ*	pBAD18-Cm containing *VPA1349* gene with *VPA1349*T and *rplL*T, and *lacZ* gene	This study

References

1. Guzman LM, Belin D, Carson MJ, Beckwith J. Tight regulation, modulation, and high-level expression by vectors containing the arabinose P_{BAD} promoter. J Bacteriol. 1995; 177: 4121–4130.
2. Pratama A, Ishii E, Kodama T, Iida T, Matsuda S. The xenogeneic silencer histone-like nucleoid-structuring protein mediates the temperature and salinity-dependent regulation of the type III secretion system 2 in *Vibrio parahaemolyticus*. J Bacteriol. 2023; 205: e0026622.
3. Okada R, Matsuda S, Iida T. *Vibrio parahaemolyticus* VtrA is a membrane-bound regulator and is activated via oligomerization. PLoS One. 2017; 12: e0187846.
4. Kodama T, Akeda Y, Kono G, Takahashi A, Imura K, Iida T, et al. The EspB protein of enterohaemorrhagic *Escherichia coli* interacts directly with alpha-catenin. Cell Microbiol. 2002; 4: 213−222.
5. Parales RE, Harwood CS. Construction and use of a new broad-host-range *lacZ* transcriptional fusion vector, pHRP309, for Gram− bacteria. Gene. 1993; 133, 23–30.
6. Gotoh K, Kodama T, Hiyoshi H, Izutsu K, Park KS, Dryselius R, Akeda Y, et al. Bile acid-induced virulence gene expression of *Vibrio parahaemolyticus* reveals a novel therapeutic potential for bile acid sequestrants. PLoS One. 2010; 5: e13365.
