# Supplementary material for: The read-through transcription-mediated autoactivation circuit for virulence regulator expression drives robust type III secretion system 2 expression in Vibrio parahaemolyticus
Source: PLoS Pathog. 2024 Mar 27;20(3):e1012094. doi: 10.1371/journal.ppat.1012094 (PMC10971746; doi:10.1371/journal.ppat.1012094)
Supplement: S4 Table — (PDF) [file ppat.1012094.s009.pdf]

S4 Table. Primers used in this study.

Primer	Sequence (5' to 3')	Description
pHRP309-Inverse-F	GAATTCCCGGGGATCCTCTA	For construction of *lacZ* reporter plasmid in ß-galactosidase assay
pHRP309-Inverse-R	GTCGACCTGCAGTTCATTCA	
SalI-up*VPA1350*-F	GGGGTCGACTTGAGAGCAATGTGGCTACTTG	
SalI-up*VPA1353*-F	TGAATGAACTGCAGGTCGACGCCAGATGCGTGAATTTGCGG	
SalI-up*VPA1356*-F	GTCGACTGATATAAGGTCAAATTAAATGAAAAAAGACTAAATCAG	
BamHI-up*VPA1356*-R	GGATCCTCAAGTTTATTCAGACTTTATCCAGC	
SalI-up*VPA1356*-F2	TGAATGAACTGCAGGTCGACTGATATAAGGTCAAATTAAATG	
SalI-*VPA1356*-F	TGAATGAACTGCAGGTCGACATGGTGAGGTGTACCTTGTATATCG	
EcoRI-up*VPA1353*-R	TAGAGGATCCCCGGGAATTCTTTTTAGCCTTTTTAAATAT	
EcoRI-up*VPA1350*-R	GGGGAATTCTTCTCCTACTTCACTTGAATC	
EcoRI-*VPA1349*stop-R	TAGAGGATCCCCGGGAATTCTTAGCCTTTTACCTCGTTAAC	
EcoRI-*vtrB* promoter (ex-35-10)-R	TAGAGGATCCCCGGGAATTCGATAAAACCGTGAAAAGCAGC	
EcoRI-*vtrB* promoter-R	TAGAGGATCCCCGGGAATTCCGCTGAGCCCTTTTCACAG	
*VPA1349-rplL*T-Inverse-F	TGATGGCTGGTGACTTTTTAGTCACCAGCCTTTTTTTCTCAAAGTTATCTTTTGAAGGTAATGGGGTATTTTTGAATAACTACATTGGGGTGTGATTG	
*VPA1349-rplL*T-Inverse-R	AAAAAGGCTGGTGACTAAAAAGTCACCAGCCATCATTAAAAATAAAAGGGGCATAGCCCCTTTAGCCTTTTACCTCGTTAACAAAATCTACAGCG	
VPA1349-HP-Inverse-F	AGGCTAAACCCCGTATGCCCCTTTTATTTTTAATTCTCAAAGTTATCTTTTGAAGGTAATGGGGTATTTTTGAATAACTACATTGGGGTGTGATTG	

S4 Table. Primers used in this study (continued).

Primer	Sequence (5' to 3')	Description
VPA1349-HP-Inverse-R	GAATTAAAAATAAAAGGGGCATACGGG GTTTAGCCTTTTACCTCGTTAACAAAAT CTACAGCGACGGCATATTGGTCATCGA CCTTAAATAATTGCCC	For construction of *lacZ* reporter plasmid in ß-galactosidase assay
Seq_*VPA1356*-*vtrB*_1F	TCATTCTAGTGACGCTGCCG	For sequencing
Seq_*VPA1356*-*vtrB*_2F	TGATGCGATCGCGGGTATTT	
Seq_*VPA1356*-*vtrB*_3F	GGACAAGCCCTTGGACTACC	
Seq_*VPA1356*-*vtrB*_4F	TGTCATATTACTGACTCCCGAACT	
Seq_*VPA1356*-*vtrB*_5F	TCTGGTGAATGGTTCTCCGC	
Seq_*VPA1356*-*vtrB*_6F	CGGAAGGCTCGCCAGAAATT	
Seq_*VPA1356*-*vtrB*_7F	AGATTTTGGGTGCGAAGAACAC	
Seq_*VPA1356*-*vtrB*_8F	GTCGTGCGCAATCGGTAAAA	
Seq_*VPA1356*-*vtrB*_9F	TGAGAGCAATGTGGCTACTTGA	
Seq_*VPA1356*-*vtrB*_10F	GCAGACTACCAGAGCAGTGG	
Seq_*VPA1356*-*vtrB*_11F	TGAAGTAGGAGAAATGACGATGCA	
Seq_*VPA1356*-*vtrB*_12F	ACACCGATTTGCAATCGTTGG	
Seq_*vtrB*_F	TTGGGGTGTGATTGTCCTCG	
Seq_downstream-*vtrB* _R	CGACTGCCATAAACATTGACTACA	
vtrB-F	TCACCCCTTCAATAAGTTGC	For qRT-PCR and northern blotting
vtrB-R	AAGCAGCGATCTTGAAATTG	
vtrB-promoter-F	CGCTGAGCCCTTTTCACAG	
vtrB-promoter-R	GGAGTTTGAAGTGGTCGTC	
downstream of *VPA1349*-F	GAATAAAGTGATGATAAAAC	
downstream of *VPA1349*-R	GTATTTTTGAATAACTACATTG	
VPA1349-F	ACCGCTTTTCAACATAACCTGA	
VPA1349-R	TGATTGCCCATTGCCAGCTA	
VPA1350-F	CTTGCGTTGGGGTGACTTCT	
VPA1350-R	CCGTAAACAGCACATTTGGGG	
vopD2-F	CTGTGTCACTGGCGGATGAT	
vopD2-R	GGCCACACGCATGTTTGAAT	

S4 Table. Primers used in this study (continued)**.**

Primer	Sequence (5' to 3')	Description
recA-F	GCTAGTAGAAAAAGCGGGTG	For qRT-PCR and northern blotting
recA-R	GCAGGTGCTTCTGGTTGAG	
vscJ2-F	TCTACCAGCACGCCATAGC	
vscJ2-R	CAGGTTGCCGTGATTCAGG	
Universal Primer (UPM)	CTAATACGACTCACTATAGGGCAAGCAGTGGTATCAACGCAGAGT	For 5'-RACE PCR
Reverse Transcriptase-*vtrB*	CTGGTAACTGAAGAGAGAAACGCAGACG	
GSP-*vtrB*	GATTACGCCAAGCTTGGCTCATCCTCCGCCACCATTAAACCT	
BamHI-*VPA1349*-*rplL*T-F1	GGGGGATCCATGAAAGTTGAATTAGATGTTGTGATTGC	For chromosomal mutant construction
VPA1349-*rplL*T-R1	GACGACCACTTCAAACTCCTAAAAAGAATTTTGCTAAATCACGGAATAAAGTGATGATAAAACCGTGAAAAGCAGCAAAAAAAACC	
VPA1349-*rplL*T-R2	CAGTTTTTCTTCGTTATTTATTGTGATTAAAAGAGAACGTCTCTGTTCTTAAAGCCGTGGTAATGAAGACGACCACTTCAAACTCCTAAAAAG	
PstI-*VPA1349*-*rplL*T-R3	CTGCAGGAGCGTATCAAAAATGACTTGGTTTCCTAGTAAGTAGTTTCTTAACATCGCTGAGCCCTTTTCACAGTTTTTCTTCGTTATTTATTGTG	
BamHI-*VPA1349*-HP-F1	GGGGGATCCCGGTAAAGATTTAGCAAATTCTAAGAG	
VPA1349-HP-R1	GTGATGATAAAACCGTGAAAAGCAGCCCAAGCTTAGCTGGCAATGGGCAATCAC	
VPA1349-HP-F2	GTGATTGCCCATTGCCAGCTAAGCTTGGGCTGCTTTTCACGGTTTTATCATCAC	
PstI-*VPA1349*-HP-R2	GGGCTGCAGGGATGTTTCACTAACAAAAACTTTAC	
