# Supplementary material for: The read-through transcription-mediated autoactivation circuit for virulence regulator expression drives robust type III secretion system 2 expression in Vibrio parahaemolyticus
Source: PLoS Pathog. 2024 Mar 27;20(3):e1012094. doi: 10.1371/journal.ppat.1012094 (PMC10971746; doi:10.1371/journal.ppat.1012094)
Supplement: Attachment — Submitted filename: Point-by-point responses.docx [file ppat.1012094.s011.docx]

**Point-by-point responses to the reviewers’ comments**

**Reviewer #1**

Major comment

In this study, the authors demonstrated that autoactivation of VtrB is induced by transcription from the operon upstream of vtrB through read-through transcription across the intrinsic terminator, which enlarged the expression of T3SS2 and the virulence of V. parahaemolyticus. The paper was written clearly and easy to understand.

I have only one comment, that is, in Figure 1d, the author only investigated the effect of overexpression of vtrA or vtrB on the promoter activity of vtrB in the ΔvtrAΔvtrB strain. The author should simultaneously test the promoter activity of vtrB in WT, ΔvtrA and ΔvtrB.

Response

As suggested by the reviewer, we performed an additional *lacZ* reporter experiment to determine the *vtrB* promoter activity in WT, Δ*vtrA*, and Δ*vtrB* strains. It should be noted that there was no significant difference in the promoter activity between WT and Δ*vtrB*, thus further supporting our notion that VtrB does not activate the *vtrB* promoter. This result is shown in Figure R1 and is now included in Figure 1D of the revised manuscript, with the additional explanation on lines 157–159 of the marked version.

**Figure R1 (included as new Figure 1D)**: β-galactosidase activity from the *lacZ* fusion reporter of the *vtrB* promoter region (P*_vtrB_*) in *V*. *parahaemolyticus* WT, ∆*vtrA*, and ∆*vtrB* strains grown under TDC induction. The values show the means, and the error bars represent S.D. (n=3). *, p < 0.05; ns, not significant, as revealed by one-way ANOVA followed by Dunnett’s multiple comparison test.

**Reviewer #2**

Major comment

The authors address how having a strong terminator between VPI1349 and vtrB decreases virulence gene expression and pathogenesis. However, this does not address the physiological role of the wild type terminator. It would be nice to see if disrupting the terminator leads to non-terminated transcripts, altered gene expression, as well as cytotoxicity.

Response

The reviewer raises an interesting point, and we have performed a new experiment in which a *V. parahaemolyticus* strain carrying the HP mutation for disrupting the hairpin structure of *VPA1349*T on the chromosome was constructed (HP strain). As shown in Figure R2, ~4,000-nt, and ~2,000-nt read-through transcripts but not ~3,000-nt, ~1,000-nt, and ~900-nt transcripts that were terminated at *VPA1349*T were observed in the HP strain with the northern blotting using the *VPA1349* probe, thus validating that the HP strain is deficient in termination at *VPA1349*T. We have also examined the expression levels of *vtrB* and T3SS2-related genes in the HP strain, and *vtrB* gene was upregulated while no significant changes in T3SS2-related genes were observed. These new data are now included in Figure S5 of the revised manuscript, and a possible inference that read-through transcription with moderate termination may optimize the amount of VtrB production is now described on lines 535–539 of the marked version.

**Figure R2 (included as new Figure S5)**: **Disrupting the hairpin structure of *VPA1349*T in the chromosome increases *vtrB* expression but not VtrB-regulated gene expression.** (**A**) *V. parahaemolyticus* strains WT, WT-derived DT (DT), ∆*vtrB*, and a strain carrying the hairpin mutation in *VPA1349*T (S3A Fig: *VPA1349*T-HP) on the chromosome of the WT strain (HP strain) were grown in LB medium containing 0.3 M NaCl to an OD600 of 0.8, and TDC was then added. After further incubation for 15 min, total RNA was extracted. Northern blotting was performed using the probe for the *VPA1349* coding region. The HP strain was defective in transcription termination at the end of the upstream operon, as observed by the absence of L and M transcripts terminated at *VPA1349*T. L, L transcript; LT, L transcript terminated at *VPA1349*T; M, M transcript; MT, M transcript terminated at *VPA1349*T. The data are representative of three independent experiments. (B) Effect of the hairpin mutation in *VPA1349*T on *vtrB* and VtrB-regulated gene expression. *V. parahaemolyticus* strains POR-2, POR-2 DT (DT), POR-2 HP (HP), and POR-2∆*vtrB* (∆*vtrB*) were grown under the inductive condition, and total RNA was extracted from each culture once the culture reached an OD_600_ of 1. Relative expression of *vtrB, vscJ2,* and *vopD2* with the housekeeping gene *recA* was analyzed by qRT–PCR. The values represent the means ± SDs from a minimum of three independent experiments. *, p < 0.05; ns, not significant, compared with POR-2 by Student’s t test. (**C**) Effect of the hairpin mutation in *VPA1349*T on the production of VtrB and T3SS2-related proteins. Bacterial whole-cell lysates and culture supernatants of *V. parahaemolyticus* POR-2, HP*,* and ∆*vtrB* strains grown under the inductive condition to an OD_600_ of 1.8 were analyzed by immunoblotting with the indicated antibodies. Whole-cell lysate proteins on the blotted membrane were visualized with Ponceau S staining for loading control (LC).

Minor comment #1

Line 61: Replace the word “pathway” with machine or apparatus. The assembled secretion system is not a pathway.

Response

As suggested by the reviewer, we changed the word “pathway” to “apparatus”, on line 61 of the marked version.

Minor comment #2

Line 376: Says “… the increase in T3SS2 expression induced by vtrB autoactivation …” given that autoactivation occurs in the wildtype strains remove the words “the increase in”. In this section the authors are really checking whether decreasing terminator read through reduces vtrB expression and therefore markers of pathogenicity.

Response

As suggested, the phrase “the increase in” has been removed on line 383 of the marked version.

Minor comment #3

Line 401: Check units for rabbit ileal loop assay. Legend says “…accumulated fluid (ml) per length (cm)…” which I take to mean ml/cm while the y-axis of the graph has cm/ml.

Response

We appreciate the reviewer pointing this out. The y-axis caption in Figure 6B has been corrected to “ml/cm”.

Minor comment #3

Line 467: The authors are discussing read through transcription but mention the translating ribosome. I am guessing this should have read RNA polymerase.

Response

Our intention there is to describe the notion that the translating ribosome at the stop codon in close proximity to a terminator can interfere with the formation of the hairpin structure of the mRNA, citing relevant references. Nevertheless, to avoid confusion we revised the description to “the translating ribosome may directly repress the hairpin folding of the terminator of the nascent mRNA” on line 475 of the marked version.
